# FDA regulations and prescription digital therapeutics: Evolving with the technologies they regulate

**DOI:** 10.3389/fdgth.2023.1086219

**Published:** 2023-04-17

**Authors:** Anthony Watson, Richard Chapman, Gigi Shafai, Yuri A. Maricich

**Affiliations:** ^1^ Regulatory Affairs, Pear Therapeutics (US), Inc., Boston, MA, United States; ^2^ Global Regulatory Affairs – Devices, Sanofi US, Cambridge, MA, United States; ^3^ Medical Affairs, Pear Therapeutics (US), Inc., Boston, MA, United States

**Keywords:** digital therapeutics, prescription digital therapeutics, food and drug administration, regulatory reform, FDA, software as a medical device (SaMD)

## Abstract

Technological progress in digital therapeutics—and, in particular prescription digital therapeutics (PDTs)—has outpaced the processes that the Food and Drug Administration (FDA) uses to regulate such products. Digital therapeutics have entered the health care ecosystem so rapidly that substantial misunderstandings exist about how they are evaluated and regulated by the FDA. This review briefly explains the relevant regulatory history of software as medical devices (SaMDs) and reviews the current regulatory landscape in which prescription and non-prescription digital therapeutics are developed and approved for use. These are important issues because PDTs, and digital therapeutics in general, are an explosively growing field in medicine and offer many advantages over conventional face-to-face treatments for the behavioral dimensions of a wide range of conditions and disease states. By allowing access to evidence-based therapies remotely and privately, digital therapeutics can reduce existing disparities in care and improve health equity. But clinicians, payers, and other healthcare stakeholders must appreciate the rigor of the regulatory frameworks within which PDTs are approved for use.

## Introduction

As in practically every other sphere of life, medicine in the past two decades has become ever more digitized. At every level, from the conduct of wholly remote digital clinical trials to the use of digital diagnostic tools and, increasingly, the use of digital therapeutics (whether prescription or non-prescription) to treat serious disease states, digital technologies are transforming healthcare. The expanded use of digital therapeutics has been fueled by the ever-increasing prevalence of chronic or difficult-to-treat conditions such as mental health conditions, substance use disorders, insomnia, and lower back pain as well as by acute shortages of providers who are skilled in delivering the behavioral therapy components of care that are so often critical to patient recovery.

But, in part because of the speed with which prescription digital therapeutics (PDTs) have entered the health care ecosystem, some uncertainty exists about how rigorously these devices are evaluated and exactly how they are currently regulated by the Food and Drug Administration (FDA). Torous et al., recently outlined how regulatory approaches can improve innovation in digital devices (1). This perspective expands on this theme, explaining the history and current regulatory landscape in which digital therapeutics are approved for use and will examine the ways the FDA is adapting to this new paradigm.

## The evolution of software regulation

The 1976 Medical Device Amendments to the Food, Drug, and Cosmetic Act requires that FDA regulate products intended to diagnose, treat, and/or manage disease. After 1987, FDA was fully aware of the potential role of computers and software in healthcare when it published its “Draft Policy on the Regulation of Computer Products.” This document provided guidelines about which software products were regulated as medical devices and which were exempt from regulatory controls such as premarket notifications. Specifically, the guidance stated that the following software was not subject to registration, listing and premarket notification (i.e., FDA authorization): (1) general purpose articles, (2) computer products manufactured by licensed practitioners for use in their practice (3) computer products used in teaching and non-clinical research, and (4) computer products which provide opportunity for competent human intervention. The guidance further stated that the following computer products would require notification to FDA prior to marketing: (1) computer products excluding competent human intervention and (2) substantially equivalent computer products. Computer products that do not meet any of the other criteria would be subject to premarket approval (see section “FDA pathways for digital technologies”). A 1989 draft policy statement, “FDA Policy for the Regulation of Computer Products,” reiterated the 1987 draft and was the agency's operational policy for almost 20 years (2).

In the years since, and particularly in the last decade, there has been a proliferation of consumer industry healthcare apps that were never considered in the original software policies. In addition, the sheer volume and diversity of the products and manufacturers has seemingly been daunting to FDA. FDA attempted to clarify the kinds of software it would regulate in the draft Mobile Medical Applications (MMA) guidance document published in 2013 and updated twice in 2019 (3). The 2019 guidance was updated to reflect the issuance of the final rule, “Medical Devices; Medical Device Classification Regulations To Conform to Medical Software Provisions in the 21st Century Cures Act” (86 FR 20278) and the guidance “Clinical Decision Support Software” (referred to as CDS guidance throughout the rest of this document) issued on September 28, 2022. This guidance excluded from regulation certain low-risk software that met the definition of a medical device, although certain quality-related activities were recommended for manufacturers.

FDA and industry seemed to be moving towards a common ground of using a risk-based approach to regulating software. Meanwhile, Congress was watching this evolution closely, and in December 2016, Congress passed the 21st Century Cures Act (4). Among other changes, the Cures Act redefined “medical device” to exclude certain types of software such as medical device data systems (e.g., a device intended to transmit, receive, display, or convert without changing, data from a medical device). Many of the software types excluded from regulation were consistent with FDA's present risk-based approach and aligned closely with the MMA Guidance. The Cures Act also clarified that FDA was not permitted to review parts of a software system that were not regulated, although the boundaries of this system would be dependent on the manufacturer's risk assessment. FDA has since published a flurry of guidance documents attempting to clarify their evolving interpretation of the Cures Act (3, 5, 6). Specifically, these guidance documents attempted to clarify (1) what types of clinical decision support the FDA would and would not regulate, (2) what types of medical device functions FDA would and would not regulate, and (3) what information FDA would expect to see in a submission for regulated medical device functions.

FDA has also been examining the way in which it works with digital health companies. The Digital Health Center of Excellence (DHCoE) was created within the Center for Devices and Radiological Health (CDRH) to lead efforts to catch up with the digital revolution (7). The DHCoE takes a strategic view of digital health devices, i.e., by working broadly with the FDA, other agencies, and external stakeholders to address regulatory approaches as opposed to simply producing guidance documents regarding specific technologies or processes related to approval or product-specific efforts.

The FDA currently regulates digitally-delivered treatments that meet the definition of software as a medical device (SaMD) (8). SaMD is defined by the International Medical Device Regulators Forum as “software intended to be used for one or more medical purposes that perform these purposes without being part of a hardware medical device” (9). The “medical purposes” include the diagnosis, mitigation, treatment, or prevention of disease. In the United States, SaMD products are primarily regulated through the traditional approaches used to approve low-to-moderate-risk medical devices (i.e., devices that pose a low-to-moderate risk of harm to patients as a result of using a device).

## FDA pathways for digital technologies

SaMD products, like all medical devices, are evaluated for their perceived potential risk to patients and are assigned to one of three classes: Class I (low risk); Class II (moderate risk); and Class III (high risk) (10). Class II devices require general regulatory controls (i.e., broad requirements for provision of information to users), and often special regulatory controls, such as a requirement for clinical data specific to a product in order to provide reasonable assurance of safety and effectiveness or to demonstrate substantial equivalence to a predicate device (11). Class III devices require general controls as well as premarket approval.

Although the first FDA-authorized PDTs were authorized as Class II devices based on their indications (requiring special controls) (11), different digital therapeutics may end up in different risk classes based on their area of treatment (12–14). The Code of Federal Regulations (CFR) lists a variety of regulations regarding computerized therapies that are unique to the diseases that a particular SaMD product is designed to treat. The regulations, therefore, are “fit for purpose.” As an example, SaMDs developed for psychiatric disorders follow the requirements for Computerized Behavioral Therapy device for psychiatric disorders (21 CFR 882.5801) (15), while SaMDs for gastrointestinal conditions are categorized and follow the requirements for Computerized Behavioral Therapy device for treating symptoms of gastrointestinal conditions (21 CFR 876.5960) (16). FDA looks not only at past decisions but considers the specific circumstances involved in each approval.

Importantly, Class II devices generally include special controls. For example, FDA might specify requirements around labeling or clinical data to satisfy questions of safety and effectiveness (11). Some existing PDT authorizations specify the requirement for subsequent products to include clinical data, which are necessary to provide reasonable assurance of safety and effectiveness (12, 13, 17–19).

Once any kind of digital treatment has been evaluated in one or more clinical studies (e.g., randomized controlled trials) the data and formal requests for authorization are submitted via one of two FDA pathways, each with regulatory and evidence-based requirements:

• The *de novo* pathway, which requires clinical data demonstrating the safety and effectiveness of the device (20). Devices authorized via this pathway can then serve as “predicates” for other devices.
• The 510(k) clearance pathway, which requires the submission of clinical data demonstrating substantial equivalence in terms of safety and effectiveness to a predicate product authorized either via the *de novo* or another 510(k) pathway (21).

Both pathways involve the submission of detailed data reports and product descriptions that inform the creation of patient and clinician labeling/instructions for use if the product is authorized.

Work is underway to create a dedicated FDA regulatory framework for SaMD products such as PDTs that reflects the unique attributes of these devices. For example, unlike pharmaceuticals, SaMD products can be frequently updated following FDA authorization, and products relying on artificial intelligence as a component of treatment may “learn” or change how their algorithms perform over time.

In 2017, the FDA announced the Software Precertification Pilot Program, which, it is hoped, will provide more streamlined and efficient regulatory oversight of software-based medical devices developed by manufacturers who have demonstrated a robust culture of quality, organizational excellence, and willingness to monitor their products once they reach the market. Nine companies have participated in the pilot program and have committed to reviewing real-world performance of their products to ensure patient safety and product quality (22).

The proposed approach looks first at the digital health technology developer, rather than solely at the product, which is the current focus of traditional medical device regulations. The new processes seek to accommodate the rapidity with which software products can respond to glitches, adverse events, and other safety concerns. In the Pre-Cert program, the FDA is proposing that software products from authorized companies would continue to meet the same safety and effectiveness standard that the agency expects for products that have followed the traditional path to market. FDA released a final report on this program in September 2022 (23). The report concluded that FDA could implement some changes under present authorities but would need legislative changes to implement others.

## Prescription digital therapeutics

Digital treatments, like other therapeutic products, may be prescription or non-prescription. Prescription products require initiation by a licensed healthcare professional, as governed by state-level health authorities. The stipulation for prescription is based on review of the product and a variety of factors by FDA. Prescriptions may be required for the treatment of serious disease, the use of higher-risk devices, the need for a secure diagnosis by a trained clinician, monitoring and follow-up to determine appropriate response, and/or to compare treatment options to determine optimal treatment approaches.

PDTs are software-based treatments delivered on smartphones or tablets that address the behavioral dimensions of many diseases and conditions (8). The first FDA-authorized PDT to make treatment claims was reSET® (to treat patients with substance use disorders) in 2017 (17, 24). This new class of therapy is expanding rapidly, in terms of coverage by payors and overall market size. In January 2022, a Research and Markets analysis valued the 2021 global market for digital therapeutics at $3.35 billion and estimated it would reach $12.1 billion by 2026 (25, 26).

While the first software-based therapeutics were PDTs, non-prescription digital treatments are similar and some of these have received FDA market authorization (14, 24). For example, the non-prescription Natural Cycles (27) software application that lets women track their menstrual cycles was approved via the *de novo* pathway as a Class II device, while another non-prescription menstruation tracker, Clue, was authorized via the 510(k) pathway using Natural Cycles as a predicate device (28).

Unlike health and wellness apps, PDTs specifically treat diseases and, therefore, are regulated by FDA and categorized as Class II devices. Although PDTs, and digital therapeutics in general, are technologically different from traditional medical devices, they are currently reviewed and authorized by CDRH using regulatory pathways and processes that have not always been aligned with the rapid, dynamic, and iterative nature of treatments delivered as software.

In some cases, PDTs may be intended to be used alongside standard of care pharmacotherapy. In such cases, such as reSET-O®, which is intended to be used alongside the pharmacotherapy buprenorphine, both FDA's CDRH *and* the Center for Drug Evaluation and Research (CDER) review and provide input, even if CDRH was the primary review center (12). We are already seeing expansion of drug/software combination products that may be regulated as drugs with CDER as the primary review center and CDRH as the consulting center.

## Patient safety, trust, and transparency for public health

FDA has recognized for decades that software is not risk free. Software can result in adverse events, mistreatment, lack of treatment, or other errors across many disease areas (29, 30). It is appropriate, therefore, that FDA regulates software that carries risk under their risk-based framework to protect public health. Organizations and stakeholders including payers, provider organizations, clinicians, and developers, have a responsibility to their patients to use products that are safe and effective. Maintaining trust and transparency is critical for patients and public health. Developers' compliance with FDA regulations and best practices is critical to maintain trust and transparency, and reduce the risk of harm. The vast majority of consumer medical apps are not regulated by FDA because these products are, presumptively, only intended to help individuals maintain general fitness, health, or wellness, and do not meet the definition of a medical device as defined above. In the authors’ opinion, it is important that FDA continue to enforce the line between regulated and unregulated products to protect patients and maintain trust for the benefit of public health (29, 30).

## Discussion

The Pre-Cert program and the Digital Health Center of Excellence mentioned previously are examples of the kinds of changes that can create FDA regulatory frameworks aligned with different product types to improve transparency, clinical responsibility, authorization efficiency, and clear labeling for stakeholders. The rise of FDA-regulated digital therapeutics has spurred regulatory evolution and provides experience to support further refinement and richness in FDA regulatory frameworks that balance risk and speed of bringing effective treatments to market, while maintaining public health. FDA, policymakers, research experts, and developers, must work together to make FDA policies related to digital therapeutics as nimble, flexible, and dynamic as the technologies themselves. However, reasonable and flexible regulation only works with responsible enforcement by FDA and compliance by the industry.
